# Extracellular Vesicles as Source of Biomarkers in Glomerulonephritis

**DOI:** 10.3390/ijms241813894

**Published:** 2023-09-09

**Authors:** Maurizio Bruschi, Giovanni Candiano, Andrea Angeletti, Francesca Lugani, Isabella Panfoli

**Affiliations:** 1 Department of Experimental Medicine (DIMES), University of Genoa, 16132 Genoa, Italy; 2 Laboratory of Molecular Nephrology, IRCCS Istituto Giannina Gaslini, 16147 Genoa, Italy; 3 Division of Nephrology and Transplantation, IRCCS Istituto Giannina Gaslini, 16147 Genoa, Italy; 4 Department of Pharmacy, School of Medical and Pharmaceutical Sciences, University of Genoa, 16148 Genoa, Italy

**Keywords:** biomarkers, extracellular vesicles, focal segmental glomerulosclerosis, IgA nephropathy, membranous nephropathy, glomerulonephritis, glomerular disease of systemic lupus erythematosus, nephrotic syndrome, proteinuria

## Abstract

Kidney disease is a global health and healthcare burden. Glomerulonephritis (Gn), both primary and secondary, is generally characterized by an inflammatory glomerular injury and may lead to end-stage renal disease. Kidney biopsy is fundamental to the diagnosis; however, kidney biopsy presents some concerns that may partly hamper the clinical process. Therefore, more accurate diagnostic tools are needed. Extracellular vesicles (EVs) are membranous vesicles released by cells and found in bodily fluids, including urine. EVs mediate intercellular signaling both in health and disease. EVs can have both harmful and cytoprotective effects in kidney diseases, especially Gn. Previous findings reported that the specific cargo of urinary EV contains an aerobic metabolic ability that may either restore the recipient cell metabolism or cause oxidative stress production. Here, we provide an overview of the most recent proteomic findings on the role of EVs in several aspects of glomerulopathies, with a focus on this metabolic and redox potential. Future studies may elucidate how the ability of EVs to interfere with aerobic metabolism and redox status can shed light on aspects of Gn etiology which have remained elusive so far.

## 1. Introduction

Kidney disease (KD) is a rapidly increasing global health and healthcare burden, with a relevant impact on national health systems, especially in low- and middle-income countries [1,2]. In a large number of cases, KD may lead to loss of kidney function, resulting in chronic kidney disease (CKD) [3]. CKD is present in around 9% of the global population, representing one of the main leading causes of death worldwide [4]. End Stage Kidney Disease (ESKD) is the final stage of CKD, defined by the need for renal replacement through dialysis or renal transplantation. Recent epidemiological studies suggested that the number of people needing renal replacement therapy is foreseen to double to 5.4 million by 2030 [5]. Hypertension and diabetes mellitus (DM) are the most common causes of CKD [2], while glomerulonephritis (Gn) represents the third leading causes of CKD [6]. Gn includes a heterogeneous group of renal disorders characterized by glomerular inflammation as a consequence of different pathogenic factors, such as autoimmunity, activation of complement cascade and other components involving both innate and the adaptive immunity. Despite its causes, Gn is histologically characterized by proliferation of renal cells (mesangial, endothelial, and epithelial cells) and by infiltration of immune cells (neutrophils, T and B cells, etc.). Therefore, different causative triggers may result in homogeneity of inflammatory responses and the Gn classifications include both causative and pathological factors. Among others, membranous nephropathy (MN), lupus nephritis (LN), and antineutrophil cytoplasmic autoantibody (ANCA)-associated Gn represent different Gn resulting from autoimmune disorders. By contrast, immunoglobulin A (IgA) membrane proliferative and post-infective Gn are characterized by renal deposition of immune complexes (IC) unrelated to autoimmune disease. Clinical manifestations of Gn, including hematuria, proteinuria, and renal impairment, are not so specific for distinguishing between different forms of glomerular involvement; usually, a kidney biopsy is needed. Kidney biopsy is fundamental to characterizing the etiology, severity, and chronicity of the glomerular lesions [7], and it is essential for a specific therapeutical approach. However, kidney biopsy may present some intrinsic limits. Without considering the procedural risks, the limited biopsy samples may not be very representative of the disease. Moreover, histological findings are common for the different forms of Gn, and it may be difficult to determine a single primary diagnosis. Therefore, more tools may be required. The nephron and the urogenital tract cells continuously release Extracellular Vesicles (EVs) [8] which are involved in renal physiology and pathology [9]. EVs is a collective term for heterogeneous lipid vesicles, which are not able to replicate that range from 30 nm to 1 μm in size released by most cells, and are present in all bodily fluids [10] as well as in tissues [11]. EVs carry a cargo of proteins, lipids, nucleic acids, and metabolites that acts as a mediator of cell-to-cell signaling in several physiological and pathological conditions [12], comprising inter-kingdom communication [13]. In recent years, the involvement of EVs in kidney disease has been a major topic of investigation [14,15,16]. Urinary EVs deeply impact the recipient cells participating in kidney development, playing a role in the physiology and pathology of the urinary tract [17]. Urinary EVs represent a valuable non-invasive source of molecular biomarkers of renal disease, as “liquid biopsy” [8]. In fact, large volumes of urine can be easily collected and analyzed [18]. In this review, we will summarize the latest progress and recent advances in the potential role of EVs in pathogenesis of Gn, with a focus on EVs’ ability to relay information on the metabolic and redox status of the parent cell.

## 2. Extracellular Vesicle Definition, Biogenesis, and Isolation

EVs have been classified into three sub-populations: exosomes (30–150 nm), microvesicles, (0.1–0.35 µm), and apoptotic bodies (0.8–5 µm) [10,19,20], each with their own biogenesis and functions. However, the updated guidelines of the International Society for Extracellular Vesicles, 2018 (MISEV2018), state that EV classification should be based on characteristics such as dimensions, density, and biogenesis, rather than on surface markers [21]. In fact, EV subtypes are heterogeneous, but often overlap in size and molecular content. Therefore, this review will use the collective term “EVs”, referring to exosomes as small (sEVs) and to microvesicles as large EVs (lEVs) [10,22]. sEVs are generated through an endocytic pathway, budding in the early endosome as intraluminal vesicles, with the participation of the endosomal sorting complex required for transport (ESCRT) [23]. Then, late endosomes (multivesicular bodies, MVBs) can either release the sEVs into the extracellular space or traffic them to lysosomes for degradation. lEVs are a heterogeneous vesicle population, originating from blebbing of the plasma membrane, which carry a cargo dependent upon the parent cell type [24]. Apoptotic bodies are large (500–4000 nm) membrane blebs formed during programmed cell death [19]. Analysis of the EV content has been conducted using high-throughput analytical techniques [25,26,27]. There is now interest in determining the topology (i.e., luminal-versus surface) of the protein EV components [10]. For a complete and detailed review of the heterogeneity of EV, refer to previous reports [25,26,27]. The whole human proteome EVs data were computed from published accessible databases such as Vesiclepedia [28] and ExoCarta [29]. EV release spans the evolutionary eras and is common to all kingdoms [13,30]. In humans, EV release is increased by inflammation, hypoxia, oxidative stress [31], and shear stress [32]. In turn, EV uptake depends on both the vesicles cargo and the recipient cell type [33], involving receptor-mediated endocytosis [20], clathrin or caveolin-mediated endocytosis, lipid raft interactions, macro-pinocytosis, and phagocytosis [33], or fusion with the membrane of the recipient cell [34]. Over the past decade, considerable progress has been made in relation to EV isolation methods [35]. Precipitation techniques, such as differential and density gradient ultracentrifugation, are currently considered the gold standard [36]. Size-based methods and affinity-based isolation methods are also applied [37]. Isolation methods are characterized by differential yield and specificity, but none of these can isolate EVs without the presence of contaminants. Commercial kits for EV isolation are also available [38]. In Table 1, we summarize the main techniques available for the EV isolation and compares their pros and cons. EVs can be analyzed via imaging techniques, such as scanning (SEM) [39], transmission (TEM), or cryo-EM electron microscopy [40,41,42]; scanning-probe microscopy (SPM); atomic force microscopy (AFM) [43]. EV size is routinely studied using nanoparticle tracking analysis [44,45,46]. The EV function is still largely debated, but there is consensus on their action in the regulation of inflammation, tumor microenvironment, and extracellular matrix modulation.

## 3. Extracellular Vesicles in Renal Disease

Urinary EVs play a dual role in KD: they can contribute to the onset or progression of the disease [89,90,91] and, on the other hand, they can be a source of disease biomarkers and bear a potential therapeutic role [92,93]. The involvement of EVs in the Gn causative mechanisms was recently reviewed [94]. More than 98% of the proteins expressed in the urinary EVs are released from cells of the urogenital tract, mainly glomerular, tubular, and bladder cells, which renders EVs a source of pathological determinants as well as of potential biomarkers [8]. Moreover, proteins are more efficiently preserved in EVs than in urine, a challenging milieu. Consistently, sEVs have been shown to concentrate biomarkers [68]. The opportunity offered by urinary EVs in the search for renal disease biomarkers was confirmed by a comprehensive proteome analysis of normal urine and of urinary EVs, showing that most of the identified proteins (1615 out of 3429 total) were contained in EVs [95]. Autosomal dominant polycystic kidney disease (ADPKD) is the most common inherited KD, due to mutations in the polycystic kidney disease 1 and 2 (*PKD1* or *PKD2*) genes [96]. EVs derived from cystic renal epithelia cells would promote cyst formation in ADPKD by lowering the amount of polycystin 1 in the cystic kidneys, affecting the biology of *PKD1* heterozygous renal epithelial cells [97]. A proteomic study of urinary EVs from subjects affected by ADPKD reported increase in periplakin, envoplakin, villin-1, and complement C3 and C9, respect to healthy controls, suggesting a possible role of EVs in the disease progression [98].

## 4. Extracellular Vesicles as Biomarker Source in Glomerular Disease

Glomerular diseases comprise distinct subsets of CKD causes that are potentially susceptible to therapy. Primary Gn encompass idiopathic nephrotic syndrome, thin basement membrane nephropathy (TBMN), Immunoglobulin A nephropathy (IgAN), Alport syndrome, and membranous nephropathy [6]. Secondary Gn, such as lupus nephritis (LN), DN, and postinfectious GN are the consequence of systemic diseases [6]. In Gn, novel biomarkers are needed, as currently kidney cellular injury is not assessed. In fact, clinical practice only follows through markers or renal functional loss, such as serum creatinine and proteinuria. The current clinical markers of disease, such as serum creatinine, or urine albumin levels, are not very sensitive and have limitations (the accuracy of creatinine levels depends on muscle mass; some patients regress to normo-albuminuria, etc.); also, they do not provide information about the cause of the renal injury and are a late sign of kidney damage. The proteins found in EVs comply with the characteristics required for a biomarker, being up/downregulated in the target population, non-invasive, readily available, and bearing diagnostic/prognostic significance (see detail in Supplementary Table S1). For example, a main target of glomerular disease is the podocyte, the specialized epithelial cell that constitutes the glomerular filtration barrier. A study set to characterize urinary EVs from patients with Gn showed that some of those expressed podocalyxin and fibroblast-specific protein 1 (FSP1), considered by the Authors as markers of their origin from podocytes [99]. In particular, FSP1 positively correlated with active glomerular injury such as biopsy-proven crescent formation [99]. To detect podocyte loss in kidney biopsies, staining for Wilms tumor factor-1 (WT-1) is utilized. Consistently, urinary sEVs may be a potential noninvasive biomarker of podocyte injury, as they express WT-1, which increases upon podocyte damage. This was the case for urinary sEVs from focal segmental glomerulosclerosis (FSGS), which encompasses a number of distinct pathological conditions that share a significant podocyte damage [100]. A major cause of ESKD in children and young adults is FSGS, histologically characterized by fibrosis and glomerular sclerosis. While primary FSGS is due to genetic mutations, in most cases secondary FSGS is idiopathic [101]. Significantly increased WT-1 expression was found in urinary sEVs from children with FSGS nephrotic syndrome [100] compared with healthy controls. The same study showed that WT-1 expression increased in urinary sEVs from an animal model of glomerulopathy prior to albuminuria [100]. WT-1 expression in urinary sEVs was also elevated in patients with Diabetic Nephropathy (DN), related to glomerular function decline, supporting the role of EV WT-1 as a biomarker of podocyte injury and of disease progression [102]. EVs play a role in renal fibrosis, the common pathological process through which all types of CKD progress to ESKD [103]. It was shown that EVs may contribute to FSGS pathogenesis and progression as EVs from FSGS patients stimulate mesangial cell proliferation and upregulate the signal transducer and activator of transcription 3 (STAT-3) pathway [104]. EVs from FSGS patients also contain information that reflects disease severity. Our first comprehensive proteomic analysis assayed mesothelial sEVs from peritoneal dialysis (PD) effluent of subjects affected by FSGS, in comparison to subjects bearing other KDs. PTP4A1 was the most statistically significant biomarker associated with PD vintage and loss of peritoneal membrane function, caused by peritoneal fibrosis, due to the unphysiological composition of PD fluids [82]. As peritoneal fibrosis is a frequent evolution of PD, which can limit the efficacy of dialytic treatment [82], it would be useful to validate PTP4A1 as a prognostic biomarker to predict the progression of renal fibrosis in PD. ANXA13, the founder member of the annexin (ANX) family, was able to distinguish with 100% accuracy sEVs from PD effluent from FSGS patients versus those without FSGS [103]. ANXA13 binds to negatively charged membrane phospholipids in a calcium-dependent manner and was shown to play a role in FSGS kidney evolution to CKD; on the other hand, ANXA13 has the potential to be a biomarker of disease evolution.

Membranous nephropathy (MN) is the leading cause of nephrotic syndrome in the adult non-diabetic population. In about 80% of cases, MN is primary. Like FSGS, MN is characterized by the absence of inflammation, and by podocyte injury. The latter is due to the deposition of IC between podocytes and the glomerular basement membrane. The search for serum antibodies against phospholipase A2 receptor (PLA2R) is utilized for MN diagnosis, with about 98% specificity. Importantly, PLA2R expression was found in sEV which represents a promising non-invasive method for diagnosing this form of MN [105]. IgAN is the most incident and prevalent primary Gn affecting both children and adults worldwide, and an important cause of ESKD [106,107]. IgAN is characterized by mesangial cell proliferation with inflammation, secondary to Ig deposition. Renal biopsy demonstrating mesangial IgA1-dominant or co-dominant deposits is needed for diagnosis. Complement C3 is also detected in most cases, and is frequently concomitant with fibrosis, as outlined in the Oxford classification [108,109]. Urinary EV protein expression displays different profiles specific to different forms of glomerular diseases. Studies in IgAN have focused on urinary sEVs, whose excretion is increased in IgAN patients compared to healthy controls or minimal change disease (MCD) subjects [110]. MCD is the most frequent cause of nephrotic syndrome, especially in younger subjects [111]. Urinary sEV chemokine ligand-2 (CCL2) expression was increased in IgAN patients compared with healthy controls [110]. IgAN clinical presentation is identical to that of thin basement membrane nephropathy (TBMN), whose clinical outcomes are less severe than that of IgAN. TBMN is usually linked to heterozygous collagen type IV alpha 3 and 4 chain (*COL4A3* or *COL4A4*) gene mutations, often representing an autosomal recessive Alport syndrome status [112]. In the absence of diagnostic biomarkers, only kidney biopsy can differentiate IgAN from TBMN. Interestingly, early IgAN could be differentiated from TBMN on the basis of the different expression in urinary sEVs of aminopeptidase-N and vasorin precursor, which are higher in TBMN, and α-1-antitrypsin and ceruloplasmin, which are higher in IgAN patients [113].

Lupus nephritis (LN) is the second most common cause of Gn, and a severe manifestation of Systemic lupus erythematosus (SLE). SLE is an autoimmune disease characterized by the glomerular presence of autoantibodies, immune complexes (ICs), and glomerular complement deposition [114]. LN causes a gradual decline in kidney function, with up to 30% of cases progressing to ESRD. The involvement of urinary EVs in LN is dual, as they may have pathogenic effects (by carrying autoantigens and contributing to complement activation and inflammation) }, but can also express protein biomarkers for diagnosing and predicting LN in SLE [115]. It was demonstrated that elevated expression of the high-mobility group box 1 molecule (HMGB1) in urinary EV plays a pathogenetic role in SLE patients with LN. In turn, the increased HMGB1 urinary EV levels render it a promising biomarker of LN in SLE [116]. EVs have both a pathogenetic role and a clinical potential for LN: urinary EVs were shown to carry a substantial part of plasma cell-free DNA (cfDNA), which may help with the management of patients with LN [117].

DN, a microvascular complication of both type 1 and type 2 diabetes mellitus, is a leading cause of Gn and CKD. DN is a chronic disease characterized by glomerular hypertrophy, proteinuria, decreased glomerular filtration, and renal fibrosis, eventually leading to ESRD. Early detection, before the onset of albuminuria, is essential. Microalbuminuria itself lacks the sensitivity to predict DN risk. The investigation of noninvasive specific biomarkers to predict DN takes advantage of EVs, as has recently been reviewed [118]. The study demonstrates that, also in the case of DN, urinary EVs play a role, but can also be used as potential biomarkers. EVs are secreted by both glomerular and endothelial cells and have been shown to play a pathogenic role in DN, possibly inducing the podocyte epithelial–mesenchymal transition, promoting fibrosis and ultimately progression to ESKD [118]. Elevated glucose plasma levels may be the stimulus that lead glomerular cells to secrete high amounts of EVs. Nonetheless, in DN, EV proteome can also serve as a reservoir of potential biomarkers of disease, as suggested by the first study on human urinary sEV proteome from DN patients, which identified a panel three proteins (alpha-1-microglobulin/bikunin precursor (AMBP), mixed-lineage leukemia protein 3 (MLL3) and voltage-dependent anion-selective channel 1 (VDAC1)) whose expression was changed in DN [119]. Considering that the clinical biomarkers for DN, such as estimated glomerular filtration rate (eGFR) and albuminuria, are insufficient in DM patients who do not have albuminuria or have DN with preserved eGFR, EV-derived biomarkers of susceptibility can hopefully be validated for the early detection of kidney dysfunction onset in DM. 

## 5. Extracellular Vesicles and Oxidative Stress

Mitochondrial dysfunction and oxidative stress are a major cause of many renal diseases [120]. Oxidative stress, resulting from inflammatory cytokine production, impaired mitochondrial function, and enhanced mitochondrial reactive oxygen species (ROS) production is involved in the pathophysiology of CKD [121]. Mitochondrial dysfunction causes impairment of metabolic and antioxidant activities, both of which are responsible for oxidative stress production and contribute to cellular damage and Gn progression. A study found that, in CKD, the mitochondrial respiratory machinery was dysregulated with oxidative stress production [122]. Notably, urinary EVs have been shown to affect important redox processes [9] and to relay bioenergetic and redox information [62,123]. Proteomic studies have shown the enriched expression of proteins clustering to aerobic metabolism on the surface of sEVs and lEVs from urine and human umbilical cord mesenchymal stem cells (HUC-MSC), which express the five mitochondrial respiratory complexes and perform oxidative phosphorylation [62,63,123]. Consistently, ExoCarta (http://www.exocarta.org) reports the expression of several subunits of the F_1_F_o_-ATP synthase and the respiratory chain complexes I to IV in the sEVs. Interestingly, the increased presence of a population of large EVs carrying mitochondrial molecules that Authors called “mitoEV” has been demonstrated to be associated with the disease activity in LN. These were shown to be IgG-coated, which would contribute to IC formation and renal damage [124]. It is tempting to presume that these “mitoEV” are the consistent subset of EVs that can carry out the oxidative phosphorylation. The lEV protein complement can recapitulate the bioenergetic and redox status of the parent cells and was proposed as tool for liquid biopsy [123]. For example, mitophagy defects were reported to be associated with glomerulosclerosis [26]. EVs from stem cells have been proposed in the therapy of renal diseases [125]. In a rat model of mesangioproliferative Gn, injection of EVs from endothelial progenitor cells was able to decrease glomerular cell injury [126]. The aerobic metabolic capacity of the EVs may exert beneficial action on Gn. EVs may be able to restore the oxidative metabolism, as was the case for cardiac injured cells [127], independently of transcriptional events. The use of mesenchymal stem cells (MSC) or MSC-derived EVs for KD repairment has been proposed, particularly for LN and DN. EVs derived from endothelial progenitor cells have been reported to exert protective effects in experimental Gn [126]. In fact, although stem cells play an immunomodulatory role and can regenerate injured tissues, they also carry a potential risk for dedifferentiation and tumorigenesis. Stem-cell-derived extracellular vesicles were shown to possess the same anti-inflammatory/immunomodulatory properties of MSC and to be able to modulate fibrosis, and delay tubular and glomerular damage, without the inherent risks [128]. There is still a paucity of clinical studies about the role of EVs in KD. Notably, MSC-sEVs have been shown to possess a bioenergetics ability that may support and restore that of an injured cell [129].

## 6. Conclusions

Since the pioneering proteomic analysis of urinary EVs of 2004 showed altered protein expression in EVs from KD patients [130], it has become clear that urinary EVs allow detection of high predictive value proteins. Some of those were not previously identified in urine, due not only to their low abundance, but also because of their origin from specific cell types, such as the podocyte, which render them useful for gathering information on the cell status. In this review, we have highlighted the double-faceted nature of EVs, whose protein cargo can be causative of the disease but may, at the same time, be a source of biomarkers of susceptibility, ideally enabling the detection of KD onset before changes in blood or invasive tests are clinically applicable. These may be represented by mutated proteins or isoforms involved in the pathogenesis of KD. For example, a different proteomic profile of urinary EVs from patients with medullary sponge kidney (MSK) and ADPKD was found, related to susceptibility to either disease. MSK is a rare nephron congenital malformation associated with cystic anomalies and nephrolithiasis [131]. Urinary EVs of ADPKD subjects was enriched in proteins involved in matrix remodeling associated with mechanisms involved in cyst formation [132]. By contrast, the proteomic profile of urinary EVs from MSK highlighted proteins associated with parenchymal calcium deposition/nephrolithiasis, and bone mineralization defects [132]. Moreover, EVs also have both harmful and cytoprotective effects in KDs. Among the latter effects is the ability of EVs to sustain the aerobic metabolic effect on the recipient cells [62]. 

## 7. Future Directions

In the foreseeable future, technological progress can pave the way for clinical application of “liquid biopsy” to provide information on Gn. Future validation studies may allow selected EV-derived biomarkers to be introduced into clinical practice for risk assessment and early diagnosis, mitigating the need for kidney biopsy in Gn. For their application in clinical nephrology, a standardization of EV extraction, storage and characterization procedures will be required. Notably, the EVs heterogeneous mixture can be further selected by applying the proper techniques to obtain discrete purified EV populations based on their surface proteins. Such an ability to isolate disease-specific EVs on the basis of the proteins they express on their surface will provide new insight into Gn diagnosis. Considering that proteins from distant anatomical sites were identified in urinary EVs, these will offer a repertoire of systemic causative biomarkers of disease. From this point of view, a systematic meta-analysis of proteomic data may help build an atlas of the proteins involved in the early stages of Gn, to detect it before the disease shows significant progression. This may help us to predict the evolution of the specific CKD towards ESRD.

## Figures and Tables

**Table 1:** Main methods currently available for extracellular vesicle (EVs) isolation.

Method	Advantage	Disadvantage	Average Size (nm)	Ref.
Ultracentrifugation	Simple procedure; reproducible; most widely used *	Specific equipment; allows us to process only six samples at the same time; high aggregation of exosomes; potential contamination with non-EVs particles such as lipoproteins.	sEV: 20–200 lEV: 1000	[10,47,48,49,50,51,52,53]
Ultracentrifugation with density gradient	Pure exosome preparation; reproducible.	Specific equipment; allows us to process only six samples at the same time; usable only for exosomes.	sEV: 20–120 lEV: 1000	[10,53,54,55,56,57,58,59,60,61,62,63]
Ultrafiltration	Simple procedure; allows us to process many samples at the same time.	Specific equipment; expensive; proteins contamination; usable only for exosomes.	sEV: 50–300 lEV: No	[10,53,55,56,64]
Size-exclusion chomatography	Pure preparation; reproducible.	Specific equipment; allows us to process only one sample at a time; usable only with small-volume samples at high EVs content; potential contamination with non-EVs particles such as lipoproteins.	sEV: 50–250 lEV: 1000	[10,51,53,64,65,66,67,68,69]
Precipitation with chemical reagents (polymers, organic solvent, etc)	Simple procedure; allows us to process many samples at the same time.	Retention of chemical compounds used in the isolation process; chemical compounds used can damage EVs function activity; potential contamination with non-EVs particles such as lipoproteins.	sEV: 50–200 lEV: 1000	[10,53,54,65,70,71,72,73,74]
Affinity (lectin, antibodies, etc.)	Pure preparation; reproducible; simple procedure; allows us to process many samples at the same time.	Expansive; eluting buffers can damage EVs function activity.	sEV: 20–120 lEV: 1000	[10,53,56,58,75,76,77,78,79,80,81,82]
Microfluidic	Pure preparation.	Specific equipment; expensive; fast only for small quantities.	Allows us to choose the size	[10,37,53,76,81,83,84,85,86,87,88]

## Data Availability

Not applicable.

## Supplementary Materials

The following supporting information can be downloaded at: https://www.mdpi.com/article/10.3390/ijms241813894/s1.
